# Compromised maternal nutritional status in early pregnancy and its relation to the birth size in young rural Indian mothers

**DOI:** 10.1186/s40795-021-00478-4

**Published:** 2021-11-17

**Authors:** Devaki Gokhale, Shobha Rao

**Affiliations:** 1 Symbiosis Institute of Health Sciences, Symbiosis International Deemed University, Pune, Maharashtra 412115 India; 2 Society for Initiatives in Nutrition and Development, Pune, Maharashtra 411007 India

**Keywords:** Low birth weight, Stunting, Sitting height, Chronic undernutrition, A young mother

## Abstract

**Background:**

Low birth weight is highly prevalent in rural India. As a chronic undernutrition problem, poor birth outcomes are closely related to various nutritional factors more prominently the poor maternal anthropometry at conception. The purpose of the study was to identify how compromised maternal nutritional status in early pregnancy affects the birth size of rural Indian mothers.

**Methods:**

It was a prospective observational study on singleton pregnant women (*n* = 204) from 14 villages in Mulshi Taluka of Pune District, Maharashtra, India. Maternal weight (Wt), height (Ht), body fat percent (BF%), head circumference (HC), and sitting height (SHT) were measured at early pregnancy (< 13 weeks of gestation) and infants’ weight and length were measured within 24 h of birth. Groups means were tested using a ‘t’ test while the trend in means was tested using ANOVA.

**Results:**

Mothers were young (21.46 ± 2.09 yrs), thin (46.46 ± 6.1 kg), short (153.39 ± 5.79 cm), and poorly nourished (19.74 ± 2.41 kg/m^2^). Mean birth weight was low (2655 ± 507 g) and prevalence of LBW and stunting at birth was highest among mothers in the lower tertile of each of the anthropometric indicators. In particular, stunting was significantly higher for mothers in lower tertile compared to higher tertile of Wt (44.6 Vs 64.6%) and was also true for HC (43.7 Vs 60.6%). Risk for LBW and stunting at birth was almost similar and was significant (*p* < 0.01) for mothers in the lower tertile of Wt, Ht, BMI, SHT, HC, and BF% as compared to those in the higher tertile of these measurements.

**Conclusion:**

All the anthropometric indicators of current undernutrition at first trimester as well as that in utero reflected by smaller HC, impose risk for LBW and stunting at birth especially among young rural mothers.

## Background

Low birth weight (LBW) is a major concern worldwide especially in a developing country like India with a prevalence of 30–35% [1]. Though the causes of LBW are few and known to be prematurity and small for gestational age the consequences are many ranging from morbidity to mortality. It is a multifactorial phenomenon of public health importance and a measure of the quality of life and survival of the future generation [2, 3]. The relationship between maternal malnutrition and its effect on birth outcome has been highlighted in several studies [4–7]. Maternal anthropometry is one such avenue wherein several indicators have been studied for their influence on birth outcome [8–11].

In particular, among various maternal anthropometric indicators maternal weight has been mostly explored in previous studies. For example, rural women from India [12], Sri Lanka [13], and Nepal [14] whose body weight was < 44.6 kg, < 50 kg, and < 45 kg respectively were shown to be high-risk mothers for delivering low birth weight babies. Though maternal height has not been studied extensively, BMI (kg/m^2^) has shown a significant influence on the risk of LBW [12, 15, 16]. Besides these indicators, maternal body fat % though measured by a few researchers [16], have rarely examined as a risk factor for LBW. Apart from the anthropometric measures several demographic variables like maternal age [17], menarcheal age [18], and parity [19] also have been studied with risk of LBW.

Unlike low birth weight, short birth length or stunting at birth has not been studied widely. Most anthropometric indicators studied are reflective of the mother’s current nutritional status rather than her past nutritional status and it is necessary to take into consideration the role of her past undernutrition too. For example, height and its components especially leg length/sitting height is known to be an indicator of past malnutrition but is never studied with risk of LBW or stunting at birth. The same is the case with maternal HC which is known as a surrogate measure of maternal undernourishment in-utero [20]. We, therefore, address these issues in the present study to examine how compromised maternal undernutrition in early pregnancy imposes a risk for LBW as well as stunting at birth.

## Methods

### Subjects

The prospective cohort study was conducted across three primary health centers (PHC) in rural areas of Mulshi Taluka, Pune, Maharashtra. These primary health centers covered 14 villages and approximately 5000 beneficiaries. The villages had a well-established network of community workers such as Anganwadi workers and ASHA (Accredited Social Health Activist) workers who were involved in door-to-door registration and tracking of newly married young women and their subsequent pregnancies. These women were referred to the nearest PHC by community health workers and hence tracking them within the first 3 months of gestation for the study in a well-established setup was possible. The sample size was determined considering the prevalence of LBW of 30%, an allowable error of 7%, a level of significance of 5%, and considering loss to follow up of 15%. Though the exact estimate was 196 we enrolled 208. Multiple pregnancies [1], stillbirths [2], and premature [1] cases were excluded from the analysis. Thus a total of 204 young (< 25 years) pregnant (< 13 weeks of gestation) women visiting the ante-natal-care facility of the PHC were recruited. At registration gestational age was determined from the self-reported date of last menstrual period (LMP) in the health care record and was confirmed using the USG reports from the PHC. The recruited women were then prospectively followed up thrice in subsequent trimesters till their delivery.

### Measurements

Maternal anthropometry was measured in duplicates by the researcher using standard tools and calibrated instruments. Weight was measured using a digital weighing balance (HBF 210 Omron Corporation) to the nearest 100 g while the woman stood barefoot and with minimal clothing. Standing height was measured using a Seca stadiometer to the nearest 0.1 cm while the woman stood barefoot. The pregnant woman was asked to sit on a table with feet off the floor comfortably placed on a stool, using a Seca non-stretchable tape sitting height was measured as the distance between the vertex and the base to the nearest 0.1 cm while being seated*.* The head circumference was measured using a Seca non-stretchable tape to the nearest 0.1 cm. The tape was placed on the widest part of the forehead and around the widest part of the back of the head while measuring. The body fat % was measured using an analyzer HBF 210 Omron Corporation validated and used in other Indian studies [21, 22] as well.

Since all the deliveries took place at the PHC the birth weight (g) and birth length (cm) of the neonate was measured after delivery within the first 24 h after birth by the health care staff. A SAT B30 weighing balance was used for these measurements. The WHO definition of low birth weight (LBW) babies i.e. birth weight less than 2500 g and that of stunting, low length (< 45 cm) [23, 24] for age was considered. All maternal and neonatal anthropometric measurements were measured in duplicates and average readings were considered for analysis.

### Questionnaire

The socio-economic and demographic information was collected through a structured questionnaire redeveloped after the focus group discussions conducted by the researchers among pregnant women, community stakeholders, and family members. The questionnaire consisted of various questions to identify their socio-economic status, demographics, and obstetric history. It was field-tested and amended after a pilot study. The questionnaire was then filled by the researcher through one-to-one interviews with the pregnant women on registration at the PHC.

### Ethical approval

The study was as per the Declaration of Helsinki and was approved by the Institutional Ethics Committee of Symbiosis International Deemed University. Written informed consent was obtained from participants voluntarily. Formal permissions from medical officers of PHC were also obtained before the initiation of the study in Mulshi Taluka.

### Statistical analysis

The measured variables were checked for normality and expressed in terms of mean ± standard deviation or percentages. Maternal anthropometric variables were divided into tertiles to assess the extent of change in lower and upper tertiles in association with birth weight and birth length. Means of groups were compared using a t-test and the linear trend was tested using one-way ANOVA. Logistic regression was used to estimate the risk (Odds Ratio) for LBW and stunting. The risks were also adjusted for maternal age but not for other socioeconomic variables as they were not found to be related. Analysis was performed using the statistical package for Social Sciences (SPSS) (version 20; SPSS Inc., Chicago. IL, USA). A *p*-value of < 0.05 was considered to be significant.

## Results

### Maternal socioeconomic status

Most of the mothers (77.5%) were young (21.46 ± 2.09 years) at conception. 77.4% had low levels (<10th standard) of school education and 42.2% were occupied as farming/laborer in addition to their household work. 42.2% of the husbands were also educated up to 10th grade and 79.9% were engaged in unskilled jobs such as driver, or owned a small shop, or worked as a clerk. A large proportion (66.5%) of mothers were married before 20 years of age and 59.3% had delayed menarche (< 14 years). Monthly income was low (< 10,000 INR or < 142 US $) in the majority (69.1%) of the families (Table 1).

**Table 1:** Socioeconomic characteristics of study participants

Variable	Category	N (%)
Family size	< 4	57 (27.9)
	4–5	33 (16.2)
	> 5	114 (55.9)
Maternal education	< 5th grade	31 (15.2)
	7th grade	39 (19.1)
	10th grade	88 (43.1)
	>12th grade	46 (22.6)
Father’s education	< 5th grade	21 (10.3)
	7th grade	28 (13.7)
	10th grade	69 (33.8)
	>12th grade	86 (42.2)
Maternal occupation	Laborer/farming	86 (42.2)
	Housewife	118 (57.8)
Fathers occupation	Laborer/farming	41 (20)
	Service	163 (79.9)
Monthly family income (US $)	< 111	58 (28.4)
	111–138	83 (40.6)
	> 138	63 (30.8)

### Maternal anthropometry

Mothers were thin (mean weight 46.46 ± 6.1 Kg), short (mean height 153.39 ± 5.79 cm) and undernourished (mean BMI 19.74 ± 2.41 Kg/m^2^). The cut-off for maternal weight for risk of LBW is given as 38 Kg and 5% of mothers in our study belonged to this category. Similarly, 4.4% of the mothers were shorter than risk cut-off for height (145 cm) and almost 33.8% of mothers were below BMI cut-off of 18.5 Kg/m^2^ indicating chronic energy deficiency. Though such risk cut-offs are not available for other anthropometric measures the Normal values have been suggested by few studies for BF% (< 30%) [25] and HC (53 cm) [26] and mean SHT (74 cm) [27] for Indian women. It can be seen that a considerable proportion of mothers have mean values below Normal for BF%, HC, and SHT (Table 2). The neonatal anthropometry indicated that the mean birth weight was 2655 ± 507 g while the birth length was 45.38 ± 4.1 cm.

**Table 2:** Mean (±SD) maternal anthropometric profile

Variable	Mean(±SD)	Risk cuts off For LBW	Percent (%) LBW below risk cut off
Weight (kg)	46.46 ± 6.1	38	5
Height (cm)	153.39 ± 5.79	145	4.4
BMI (kg/m^2^)	19.74 ± 2.41	18.5	33.8
BF (%)	25.34 ± 4.71	–	
HC (cm)	52.51 ± 1.39	–	
SHT (cm)	74.64 ± 2.8	–	

*BMI* Body Mass Index, *BF%* Body fat %, *HC* Head circumference, *SHT* sitting height

### Maternal anthropometry and prevalence of LBW

The overall prevalence of LBW was 27.5% and that for stunting at birth was even larger viz. 38.2%. Mean neonatal birth weight and birth length were examined by tertiles of various maternal anthropometric indicators (Table 3). Mean birth weight and length both increased significantly (*p* < 0.05) from lower tertile to higher tertile for all maternal nutritional status indicators. The difference in the mean birth weights and lengths between lowest and highest tertile of maternal weight was highest (464 g; 3.02 cm respectively; *p* < 0.001 for both). The prevalence of LBW and that of stunting at birth were highest for mothers in the lower tertile and decreased significantly (*p* < 0.05, for both) from lower to higher tertile for most indicators. It was interesting to note that the prevalence of stunting at birth was higher than that for LBW in the case of each indicator. In particular, the prevalence of stunting was significantly higher than that for LBW, for lower tertile of maternal weight (44.6 Vs 64.6%; *p* < 0.01) and smaller head circumference (43.7 Vs 60.6%; *p* < 0.01) highlighting the importance of both current and past maternal undernutrition. Additionally, it is worthwhile to note that the prevalence of stunting at birth is over 30% even in higher tertile of maternal height as well as sitting height as both are reflective of the mother’s past undernutrition (Table 3).

**Table 3:** Mean (±SD) neonatal measures by tertiles of maternal anthropometric indicators

Indicators	Tertiles (value)	N	Birth weight(g)	Birth length (cm)	%LBW	% Stunting	
Weight (kg)	Lower (< 42.26)	65	2391 ± 463	43.64 ± 3.7	44.6	64.6	
	Middle (42.26–48.73)	72	2706 ± 440	45.77 ± 4	20.8	31.9	
	Higher (> 48.73)	67	2855 ± 510	46.66 ± 4	17.9	19.4	
	p trend		< 0.001**	< 0.001**	0.001**	< 0.001**	
Height (cm)	Lower (< 150.10)	68	2506 ± 462	44.20 ± 4	34.8	53	
	Middle (150.10–155.40)	66	2674 ± 521	45.94 ± 4	29.4	29.4	
	Higher (> 155.40)	70	2777 ± 504	45.96 ± 4.1	18.6	32.9	
	p trend		0.002*	0.013*	NS	0.010**	
BMI (kg/m^2^)	Lower (< 18.5)	69	2466 ± 415	44.20 ± 3.4	39.1	58	
	Middle (18.5–20.69)	68	2655 ± 518	45.25 ± 4.4	26.5	36.8	
	High (> 20.69)	67	2849 ± 513	46.74 ± 4.1	16.4	19.4	
	p trend		< 0.001**	< 0.001**	0.012**	< 0.001**	
BF %	Lower (< 22.73)	66	2414 ± 497	44.07 ± 4.1	47.8	56.7	
	Middle (22.73–27.86)	67	2671 ± 433	45.15 ± 4	19.7	37.9	
	Higher (> 27.86)	71	2867 ± 486	46.83 ± 3.8	15.5	21.1	
	p trend		< 0.001**	< 0.001**	< 0.001**	< 0.001**	
SHT (cm)	Lower (< 70)	64	2522 ± 529	44.72 ± 4.4	42.2	45.3	
	Middle (70–75.43)	68	2700 ± 525	45.20 ± 4.3	23.5	39.7	
	Higher (> 75.43)	74	2730 ± 450	46.14 ± 3.5	18.1	30.6	
	p trend		0.018**	< 0.001**	0.005**	NS	
HC (cm)	Lower (< 52.16)	71	2410 ± 421	43.73 ± 3.9	43.7	60.6	
	Middle (52.16–53.2)	58	2719 ± 548	45.88 ± 3.9	19	29.3	
	Higher (> 53.2)	75	2837 ± 517	46.56 ± 3.9	18	24	
	p trend		< 0.001**	< 0.001**	0.001**	< 0.001**	

One way ANOVA; *p value < 0.05; **p value < 0.01

*BMI* Body Mass Index, *BF%* Body fat %, *SHT* Sitting height, *HC* Head circumference

### Risk assessment

The risk of LBW and stunting at birth by maternal nutritional status indicators was assessed using logistic regression and higher tertile as a reference category for each indicator. It can be seen that the risk for LBW was three times higher for mothers with lower tertile of weight, height, BMI, BF%, HC, and SHT. The risk of stunting at birth was larger than the risk for LBW in the case of almost all the indicators. However, it appeared more discriminatory unlike risk for LBW. It was highest (OR 7.5; CI:3.4–16.7) for lower maternal weight followed by lower BMI (OR 5.7; CI:2.6–12.3), smaller HC (OR 4.8, CI:2.3–10.3), and lower BF% (OR 4.8; CI: 2.3–9.9).

These findings remained unchanged even after adjusting both the risks for maternal age suggesting an independent influence of maternal nutritional status on neonatal anthropometry. Thus, our observations report for the first time the effect of compromised maternal nutritional status on the risk of stunting at birth in addition to the risk of LBW. Additionally, our findings highlight the importance of the mother’s past undernutrition as reflected by smaller HC and short height and sitting height in addition to current undernutrition (Table 4).

**Table 4:** Risk of LBW and stunting at birth by maternal anthropometric indicators

Indicators	Tertile	OR (95% CI) for LBW		OR (95% CI) for Stunting	
		Unadjusted	Adjusted^+^	Unadjusted	Adjusted ^+^
Weight (kg)	> 48.73	1	1	1	1
	42.26–48.73	1.2 (0.5–2.8)	1.1 (0.4–2.7)	1.9 (0.8–4.2)	1.8 (0.8–4)
	< 42.26	3.6 (1.6–8.1) **	3.5 (1.5–7.9)*	7.5 (3.4–16.7)**	6.8 (3–15.2)**
Height (cm)	> 155.40	1	1	1	1
	150.10–55.40	1.8 (0.8–4.0)	1.8 (0.8–4.1)	0.8 (0.4–1.7)	0.8 (0.4–1.7)
	< 150.10	2.3 (1–5.1)*	2.3 (1–5.1)*	2.3 (1.1–4.6)**	2.2 (1.1–4.6)*
BMI (kg/m2)	> 20.69	1	1	1	1
	18.5–20.69	1.8 (0.7–4.2)	1.7 (0.7–4.1)	2.4 (1.1–5.2)	2.2 (1–5)*
	< 18.5	3.2 (1.4–7.3) *	3.1 (1.3–7)**	5.7 (2.6–12.3)*	5.1 (2.3–11.3)**
BF%	> 27.86	1	1	1	1
	22.73–27.86	1.3 (0.5–3.2)	1.2 (0.5–3.1)	2.2 (1–4.8)*	2 (0.9–4.3)
	< 22.73	4.9 (2.2–11.1) **	4.7 (2.1–10.9)**	4.8 (2.3–10.3)*	4.3 (2–9.2)**
SHT (cm)	> 75.43	1	1	1	1
	70–75.43	1.3 (0.6–3.1)	1.3 (0.5–3)	1.4 (0.7–3.0)	1.4 (0.7–2.8)
	< 70	3.3 (1.5–7.2)*	3.2 (1.4–7)**	1.8 (0.9–3.8)*	1.7 (0.8–3.5)
HC (cm)	> 53.2	1	1	1	1
	52.16–53.2	1.0 (0.4–2.4)	0.9 (0.3–2.3)	1.3 (0.6–2.8)	1.1 (0.5–2.5)
	< 52.16	3.3 (1.6–7.1)**	3.1 (1.4–6.7)**	4.8 (2.3–9.9)**	4.4 (2.1–9.1)**

Odds ratio; *p value < 0.05; **p value < 0.01; ^*+*^ *Adjusted for maternal age*

*BMI* Body Mass Index, *BF%* Body fat %, *SHT* Sitting height, *HC* Head circumference

## Discussion

Maternal nutritional status has been considered to be the most important factor influencing birth outcomes. LBW due to poor pre-pregnancy weight [28, 29], poor gestational weight gain [8, 28], short stature [13, 30] and poor arm circumferences [13, 31] have been studied globally. Maternal HC and SHT are of prominence as they reflect the past nutritional status of the mother. About stunting at birth and associated maternal factors there is hardly any research evidence documented. We observed that apart from the current undernutrition, maternal past undernutrition reflected by smaller HC, shorter height, and shorter sitting height also confer risk for both LBW and stunting at birth. The risk is doubled for stunting at birth than LBW significantly for the lower maternal weight (< 42.26 kg) and smaller HC (< 52.16 cm) which often is unnoticed.

The mothers in our study were overtly undernourished with poor maternal anthropometry. 33.5% of mothers were chronically undernourished (BMI < 18.5 kg/m^2).^ These observations are similar to those reported by Raje et al., 2015 [32] among rural women from Maharashtra. Poor maternal BMI is known to be one of the major risk factors for poor birth outcomes. In our study too, the mean birth weight was low (2655 ± 507 g), with the shorter birth length.

(45.38 ± 4.1 cm). The prevalence of LBW was 27.5% which was similar to that reported for South Asia (UNICEF global database 2014) and was closer (24.7%) to the region-specific western rural populations of India [33]. The prevalence of stunting at birth was even higher (38.2%) than that for LBW but none of the Indian studies have previously reported.

Several studies have reported a positive correlation between maternal anthropometry and mean birth weights in different populations [34–36]. India’s poor fetal growth is at least partly caused by a maternal chronic energy deficiency and stunting [37–39]. Both mean birth weight and length at birth increased as maternal nutritional status improved from lower tertile to higher tertile for each indicator. Differences observed in mean birth weight for babies of mothers in the lower and higher tertile were similar to those reported from a study in rural areas of Maharashtra [21]. However, there aren’t any studies reporting the association of maternal HC with that of stunting at birth.

The highest prevalence of stunting at birth was observed in the lower tertile of maternal weight, the indicator of current undernutrition, as well as among mothers in the lowest tertile of HC, the indicator of maternal undernutrition in utero. These observations highlight the enormous intergenerational impact of compromised maternal nutritional status more on stunting at birth than that of LBW and hence deserves further attention.

Although, the factors associated with the birth outcome are well studied those associated with risk of LBW or stunting at birth are not [40, 41]. Identifying nutritional factors associated with these risks are of critical importance as achievement in reduction of prevalence will have multifactorial benefits such as reduction in mortality and morbidity, and improving the growth rate of children, etc. We observed that the risk for LBW was three times higher for mothers in the lower tertiles for Wt, Ht, BMI, BF, HC, and SHT compared to those in the higher tertile. Similar risks are reported for women from Ethiopia [42], Pakistan [43], and Maharashtra [44]. However, these studies examined risk cut-offs for height, weight, and BMI but not for SHT and HC. In the event of scarce data, it may be noted that our observations offer risk cut-offs for maternal SHT (70 cm) and HC (52 cm) for early detection of the risk of LBW and stunting at birth.

Although maternal nutritional status indicators such as shorter sitting height, smaller head circumference have shown to be associated with LBW [ 12], their association with stunting at birth has not been explored. We observed that the risk of stunting at birth was much higher than that for LBW, especially in the case of maternal weight (OR = 7.5; CI: 3.4–16.7), BMI (OR = 5.7; CI: 2.6–12.3), and HC (OR = 4.8; CI: 2.3–9.9). It is worthwhile to note that the risks remained significant even after adjusting for maternal age indicating an independent influence of compromised maternal nutritional status.

We would like to mention some of the limitations. Firstly, the study does not address other factors such as physical activity and diet which are also known to affect the birth outcome. Because all women came from farming families with similar socio-economic backgrounds it is unlikely to affect the associations of maternal anthropometry with birth outcome observed in this study. Maternal diet being a major avenue will be dealt with in another communication and cannot be accommodated in the same paper. Other factors such as substance abuse are not culturally acceptable and hence have not been considered.

## Conclusion

The observation in this study that smaller SHT, like smaller maternal HC, seems to be a surrogate measure of maternal undernutrition in utero and deserves attention considering the Barker hypothesis for risk of metabolic diseases in later life [45, 46]. The possible physiological explanation appears to be that maternal undernutrition prioritizes the blood flow towards the protection of vital organs such as the brain and heart situated in the upper part of the body affecting skeletal growth of epiphysis [47] probably resulting in a shorter sitting height. The intergenerational influence of smaller maternal size is documented by Ramakrishnan U et.al, 1999 [48] in the Guatemalan birth cohort. Future studies can be conducted and validated in similar settings of other developing countries in South East Asia.

## Data Availability

The datasets used and analyzed for the present study will be available from the corresponding author on reasonable request.

## Abbreviations

LBW: Low Birth Weight
OR: Odds ratio
AOR: Adjusted Odds Ratio
CI: Confidence Interval
SPSS: Statistical Package for Social Sciences
